# Genome-wide identification, phylogeny and expression analysis of the PME and PMEI gene families in maize

**DOI:** 10.1038/s41598-019-56254-9

**Published:** 2019-12-27

**Authors:** Panpan Zhang, Hao Wang, Xiner Qin, Kuan Chen, Jiuran Zhao, Yanxin Zhao, Bing Yue

**Affiliations:** 1 National Key Laboratory of Crop Genetic Improvement, Huazhong Agricultural University, Wuhan, 430070 China; 2 Beijing Key Laboratory of Maize DNA Fingerprinting and Molecular Breeding, Maize Research Center, Beijing Academy of Agriculture and Forestry Sciences, Beijing, 100097 China

**Keywords:** Phylogenetics, Evolution

## Abstract

Pectins, the major components of cell walls in plants, are synthesized and secreted to cell walls as highly methyl-esterified polymers and then demethyl-esterified by pectin methylesterases (PMEs). The PMEs are spatially regulated by pectin methylesterase inhibitors (PMEIs). In this study, 43 and 49 putative *PME* and *PMEI* genes were identified in maize, respectively. Gene structure and motif analysis revealed that members in the same paralogous pairs or in the same subgroup generally had common motif compositions and gene structure patterns, which indicates functional similarity between the closely related *ZmPME*/*PMEI* genes. Gene ontology annotation analysis showed that most of the *ZmPME*/*PMEI* genes are involved in cell wall modification and pectin catabolic process with molecular functions of pectinesterase or pectinesterase inhibitor activities. There are 35 *ZmPME*/*PMEI* genes expressed higher in anthers than in other tissues from the NimbleGen maize microarray data, and the semiq-RT-PCR assay revealed most of these ZmPME/PMEIs specially expressed in anthers and pollens, indicating they possibly had role in anther and pollen development. In addition, these *ZmPME*/*PMEI* genes were highly expressed in the fertile anthers, while lowly or no expressed in sterile anthers. This further indicated these genes might be involved in the development of anther and pollen.

## Introduction

Pectins, which are synthesized from nucleotide sugars, are the major components of cell walls in plants^1–4^. PME and PMEI play a central role in the synthesis and metabolism of pectins^5,6^. There are 66 PMEs in *Arabidopsis*^7^, 16 in *Phytophthora sojae*^8^, 35 in rice^9^, 105 in flax^10^, and 81 in *G. raimondii*^11^. For the PMEIs, 71, 49, 95 and 100 PMEIs were identified in the whole genomes of *Arabidopsis*^12^, rice^13^, flax^10^ and *B*. *campestris*^14^, respectively.

PMEs (EC. 3.1.1.11) are enzymes belonging to the class 8 of the carbohydrate esterases^15^ (CAZY: http://www.afmb.cnrs-mrs.fr/CAZY/). The mature and active region of *PME* genes mainly consists of the PME domain. In higher plants, the *PME* genes are classified into two types, type I and type II. They share a catalytic PME domain at the C-terminus, and proteins in type I also have a domain (PRO-region) at the N-terminal region sharing similarities with the PMEI domain, which demonstrated roles on early demethylesterification of pectins in the Golgi apparatus^16–18^. The PMEs catalyze the demethylesterification of homogalacturonan component of pectin, which generates carboxyl groups during the release of methanol and hydrogen ions^19^. This enzymatic activity of the PMEs can lead either to cell wall loosening or to cell wall stiffening, depending on the apoplastic pH^6,19,20^, which is sometimes associated with growth^21^, and cell-to-cell cohesion^22^. Pectin demethylesterification is catalyzed by a number of the PMEs isoenzymes which can express their activities in response to certain developmental or environmental cues and/or in a tissue-specific fashion. For example, while some PMEs are ubiquitously present^23^, others are specifically expressed during root development^22^, fruit ripening^24,25^, or stem elongation^26,27^. Analysis of pollen-specific transcriptome of *Arabidopsis* indicated that several PMEs are specifically expressed in floral buds^28^. Furthermore, in *Arabidopsis* some *PME* genes (*At5g49180*, *At1g11590* and *At4g0*2*300*) might be involved in the early event of embryo/seed development^7^. The PMEIs, first identified in kiwi^5,29^, typically inhibit the PMEs of plant origin by covering the shallow cleft of the PMEs and forming a reversible stoichiometric 1:1 protein complex^30^. Post-translational regulation of the PMEs via PMEIs represents another important control mechanism^29^. For example, *OsPMEI28* overexpression in rice had an effect on the growth process, which resulted in a dwarfed phenotype^31^, and overexpression of the *PMEI5* resulted in a higher demethylesterification of seeds and reduced the PME activity, which was accompanied by an earlier and faster germination process compared to wildtype in *Arabidopsis*^32^.

In recent years, many reports have shown that some PME/PMEIs regulate plant stress resistance and pollen development. *AtPMEI10*, *AtPMEI11* and *AtPMEI12* were identified as upregulated in response to *B. cinerea* infection^33^. Expression profile of the genes *TaPME21-2*, *TaPME21-1*/*2*/4, *TaPME58*, *TaPME63* and *TaPME67* was induced in the susceptible cv. Bobwhite and repressed in the resistant cv. Sumai 3^34^. The transgenic rice overexpressing *OsPME14* showed higher PME activity and Al content in root tip cell wall, and became more sensitive to Al stress^9^. In flax, 48 (77.4%) *PME* genes and 53 (80.3%) *PMEI* genes had higher expression level in the flowers^10^. In *Arabidopsis*, 15 PMEs were highly expressed in pollen and 10 of these contained PRO regions^35^. These suggest that the PME/PMEIs might play important roles in pollen development. Mutations of *VANGUARD1* (*VGD1*), the type I *PME* gene with the highest expression levels in *Arabidopsis* pollen tubes, resulted in retarded growth in the style and transmitting tract and subsequent reduction in male fertility^36^. In maize, the *ZmC5* of PMEs has a role in pollen tube elongation^37^ and *ZmGa1P*, a pollen-expressed *PME* gene, can confer the male function in the maize unilateral cross-incompatibility (UCI) system^38^.

In this study, genome-wide identification of *ZmPME*/*PMEI* genes was firstly conducted in maize, and the phylogenetic tree, gene structure, conservative motif, expression, gene ontology annotations were also examined. In addition, semiq-RT-PCR assay was conducted to verify the gene expression pattern of some *ZmPME*/*PMEI* genes highly expressed in anthers, since more than half the genes highly expressed in anthers according to the NimbleGen maize microarray data. To further evaluate their possible roles on pollen development, gene expression of some *ZmPME*/*PMEI* genes in fertile and sterile anthers was also investigated. Results in this study would provide useful information for further investigate the function of maize PME/PMEIs, especially on the development of anther and pollen.

## Results

### Identification of *ZmPME*/*PMEI* genes in maize

**Figure 1:**
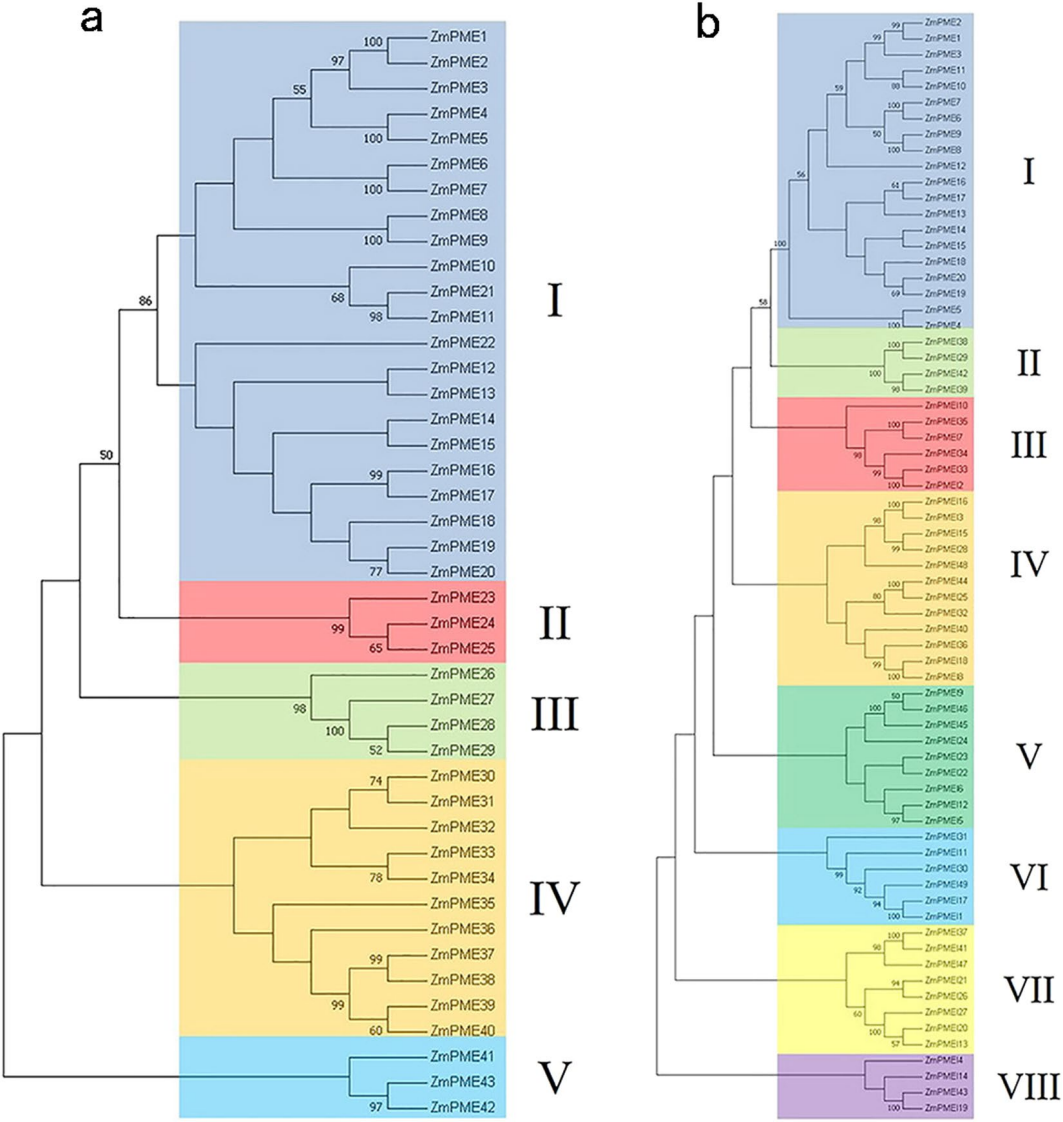
Phylogeny of the PME/PMEIs in maize. (**a**) *ZmPME* genes are clustered into 5 subfamilies. (**b**) The *ZmPMEI* genes are clustered into 8 subfamilies. The neighbor-joining (NJ) phylogenetic tree of the *ZmPME*/*PMEI* genes was constructed based on the amino acid sequence alignment of the proteins. Bootstrap values > 50% are indicated at each node. The neighbor-joining (NJ) phylogenetic trees of the ZmPME/PMEIs were built by MEGA7 (https://www.megasoftware.net/).

To identify putative *ZmPME*/*PMEI* genes in maize genome, we searched the maize genome annotation data with known plant PME/PMEI domains (pfam01095/pfam04043) as a query using HMMER 3.0 package^39^. In total, we obtained 43 putative *PME* genes and 49 putative *PMEI* genes in maize. These genes were designated as *ZmPME1*-43 and *ZmPMEI1*-49 (Fig. 1 and Supplementary Table 1), of them, 20 genes (*PME1*-20) had PRO region (which showed similarities with the PMEI domain) and the PME domain. Each *ZmPME*/*PMEI* gene model was selected by analyzing the similarity between the *ZmPME*/*PMEI* genes and homologous genes, as most of the *ZmPME*/*PMEI* genes had more than one transcript in the MaizeGDB database (https://www.maizegdb.org/). Then we randomly selected 15 *ZmPME*/*PMEIs* for reverse transcription polymerase chain reaction (RT-PCR) to assess the veracity of the *ZmPME*/*PMEI* genes models. The results indicated that the 15 *ZmPME*/*PMEI* genes were expressed in maize pollen and only a single amplicon was found (Supplementary Fig. S1). The most identified *ZmPME*/*PMEI* genes encode proteins with 150-250 amino acids (aa). They are ranging from 149 (*ZmPME23*) to 1,360 (*ZmPME28*) aa, with an average of 346 aa (Supplementary Fig. S4), and their isoelectric points (pI) are 4.28 to 10.23. These ZmPME/PMEIs are distributed on all the 10 maize chromosomes, and chromosomes 1, 2, 3, 7 and 8 have more ZmPME/PMEIs than others (Supplementary Fig. S2).

### Phylogenetic analysis

Phylogenetic trees were constructed by using MEGA 7.0 with the neighbor-joining model. In order to analyze the evolutionary relationships among the predicted ZmPMEs and ZmPMEIs, we aligned maize acid sequences with 101 and 106 predicted PMEs and PMEIs from rice and *Arabidopsis*. On the basis of phylogeny, the PMEs and PMEIs families in plants were subdivided into 5 and 12 groups, respectively (Supplementary Fig. S3). PMEs in each group and PMEIs in groups I to III, V, VI and VIII are all from the three species (Supplementary Fig. S3), indicating that these ZmPME/PMEIs might have the conserved function in evolution.

**Figure 2:**
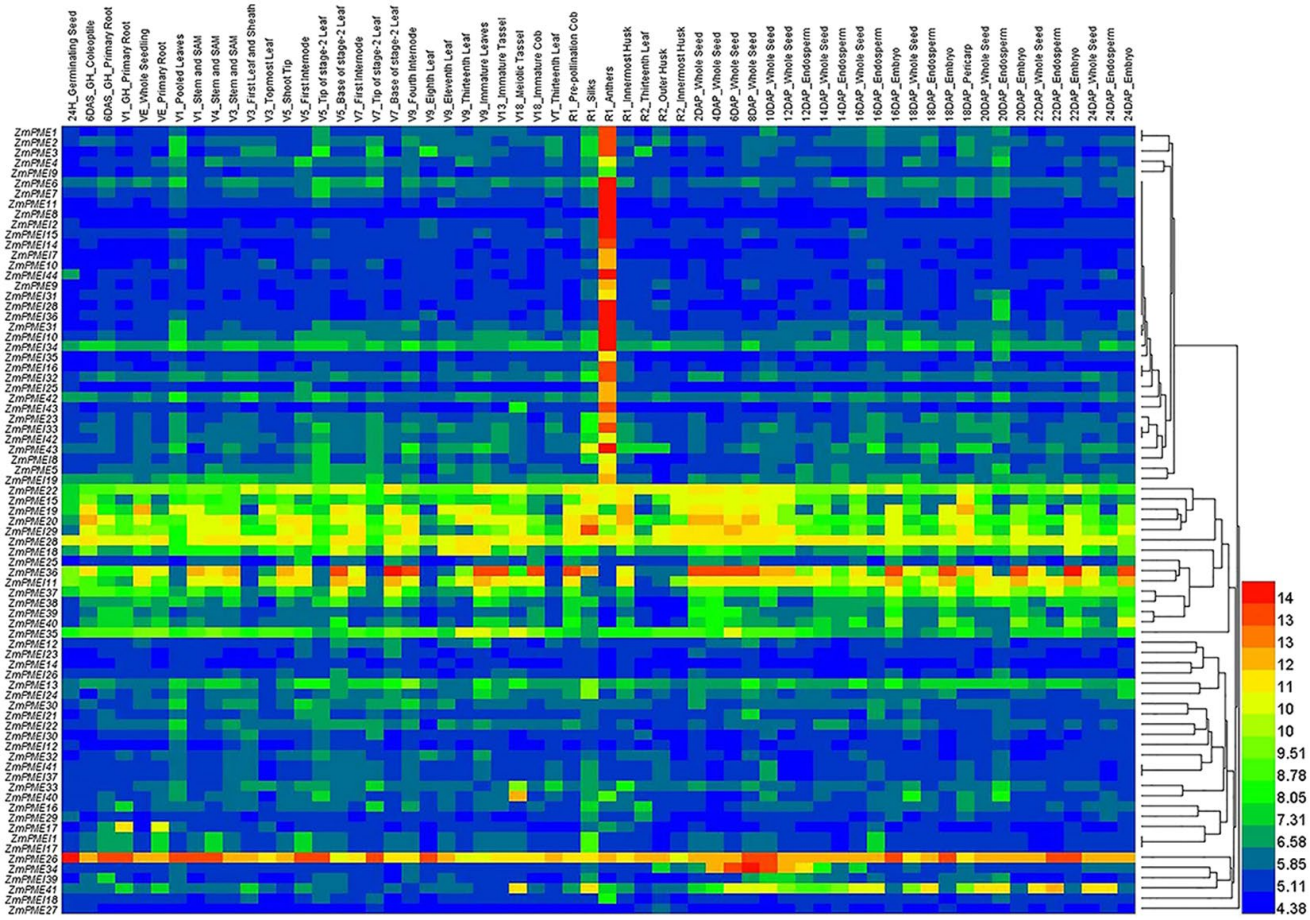
Gene expression profile of the *ZmPME/PMEI* genes in different tissues. The expression data of the *ZmPME*/*PMEI* genes was download from PLEXdb (http://www.plexdb.org/) and the heat map of the *ZmPME*/*PMEI* genes expression was generated by Heml1.0.3.7-Heatmap illustrator (http://ccd.biocuckoo.org/).

Meanwhile, according to cluster analysis, the ZmPME/PMEI families could be divided into 5 and 8 subfamilies, respectively (Fig. 1). The PMEI domains may be derived from duplication and divergence of the PRO domain and have rapidly evolved^12^. So, we constructed the PMEI phylogenetic tree used the protein sequences of all the ZmPMEIs and the 20 ZmPMEs containing PRO region. The ZmPMEI subfamily I includes the same genes in the ZmPME subfamily I (except *ZmPME21* and *ZmPME22*), both of the subfamilies are the largest subfamily, and the genes had signal prediction or transmembrane region domain. For ZmPMEs, genes in the subfamilies II and III do not have signal prediction or transmembrane region domain (except *ZmPME27*); while genes in the subfamily IV have signal prediction domain (except *ZmPME32* and *-35*); and the pI of subfamily V are higher than 8. For ZmPMEIs, genes in the subfamilies II and VII have signal prediction domain, and their pI are higher than 7 (except *ZmPMEI26*, -37 and -41); most genes in the subfamilies III, IV and VIII expressed higher in anthers than in other tissues (Fig. 2). Homologous *ZmPME*/*PMEI* genes were identified 24 paralogous pairs in maize (Supplementary Table 2). The value of the nonsynonymous substitution rate (Ka) to the synonymous substitution rate (Ks) substitutions (Ka/Ks) can be used as an indicator which could reflect selection pressure of a gene or a gene region during evolution. To infer the influence of selection on the evolution of the maize, we estimated Ka/Ks values for all of them (Supplementary Table 2). The Ka/Ks values of all the homologous genes are between 0.0033 and 0.3889, suggesting that most of the *ZmPME*/*PMEI* genes undergone negative selection and evolved slowly. The Ka/Ks values of maize PMEs paralogs are significantly lower than that of the PMEIs homologs (*P* < 0.005).

### Gene structure and motif analysis of the ZmPME/PMEI families

Gene structures of the *ZmPME*/*PMEI* genes were constructed by aligning the extracted genomic sequences to predicted cDNA sequences of the maize *PME*/*PMEI* genes. As can be seen from Supplementary Fig. S5, most of the *ZmPME* genes in subfamily I have 2 exons, and the ZmPME members in the subfamily III have 5 introns (except *ZmPME28*). In addition, most of the *ZmPME* genes contain 1–10 introns, and most of the *ZmPMEI* genes do not have no intron.

Analysis of the ZmPME/PMEI protein sequences with MEME (http://meme-suite.org/tools/meme) revealed 6 conserved motifs of the *ZmPME* genes, and 9 conserved motifs of the *ZmPMEI* genes (Supplementary Table 3). Of the ZmPMEs, 36 proteins contain motifs 1, 2, 4, 5 and 6 (except *ZmPME21*, -23, -24, -25, -27, -29, -32, -34 and -43, Supplementary Fig. S6). For the ZmPMEIs, the proteins in the subfamily I (containing both PME and PMEI domains) have motifs 1 to 9 (except *ZmPM13* to *ZmPME16*), and the rest of ZmPMEIs contain motifs 8 and 9 (except a few *ZmPMEI* genes, Supplementary Fig. S7).

**Figure 3:**
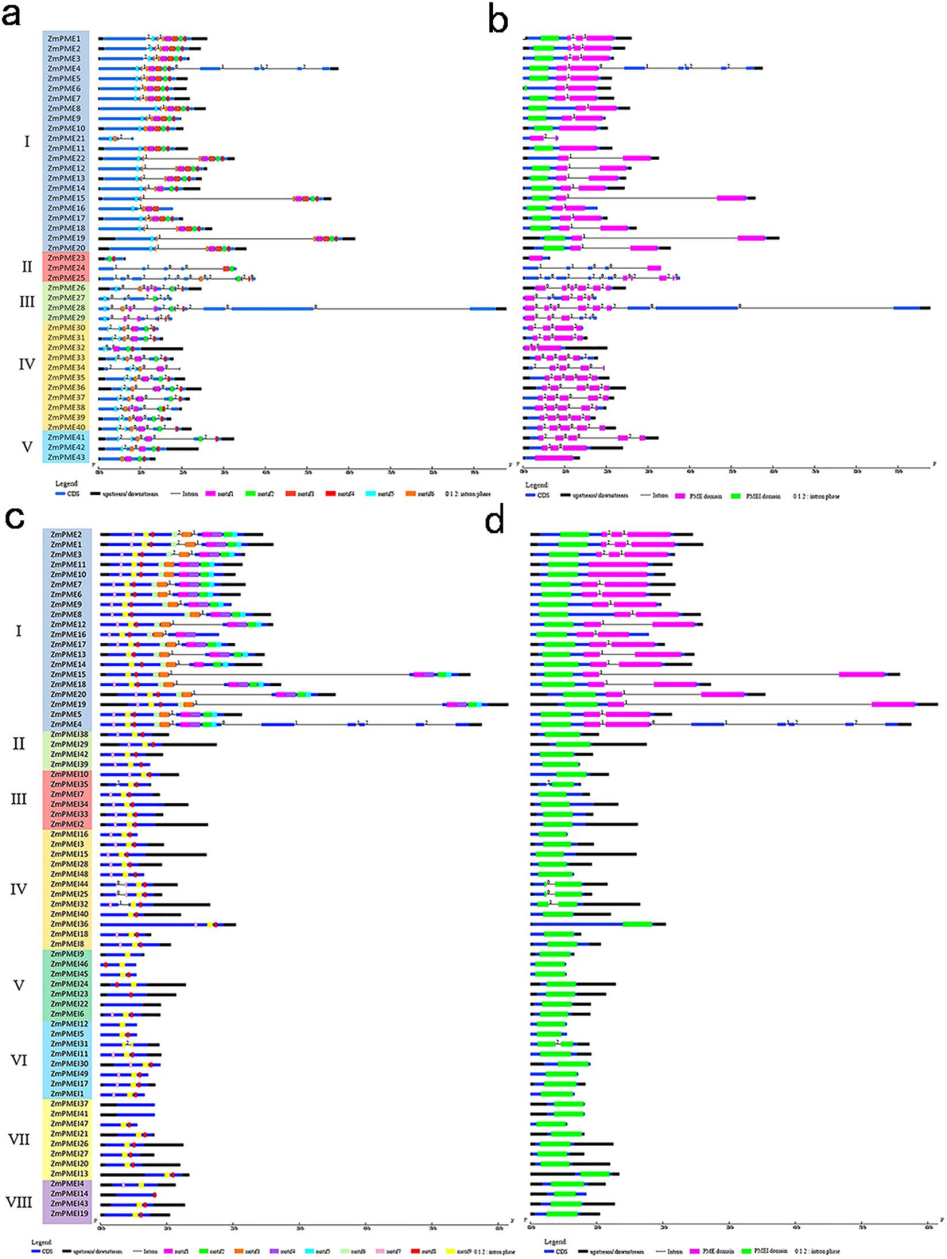
Conserved gene structures of the *ZmPME*/*PMEI* genes. (**a,b**) conserved motifs and domains of ZmPMEs. (**c,d**) conserved motifs and domains of ZmPMEIs. The gene structures of the *ZmPME*/*PMEI* genes was built using GSDS2.0 (http://gsds.cbi.pku.edu.cn/index.php) through both alignment of DNA obtained from MaizeGDB (http://www.maizegdb.org/), coding sequences (CDS) obtained from ORF finder (https://www.ncbi.nlm.nih.gov/orffinder/) and ZmPME/PMEI domains obtained from Pfam (http://pfam.janelia.org/) or SMART (http://smart.embl-heidelberg.de/) of the *ZmPME*/*PMEI* genes.

As expected, most closely related members have a common motif composition and gene structure pattern, which indicates functional similarity between the ZmPME/PMEI proteins in paralogous pairs or in the same subfamily (Fig. 3). For the *ZmPME* genes, proteins in the subfamilies IV and V contain motifs 1–5 (except *ZmPME32*, -34 and -43) and the intron phase 2, 0, 0 and 2 separating the PME domain (except *ZmPME32*, Fig. 3a,b). Proteins in the subfamily VII of the ZmPMEIs have motifs 8 and 9 (except *ZmPMEI37* and -41), while that in the subfamilies II, III and IV have motifs 7, 8 and 9 (except *ZmPMEI35* and -40, Fig. 3c,d), and most of *ZmPME* genes in the subfamily IV have 5 exons.

The PME and PMEI domains of the ZmPME/PMEIs, the PME domains in *E. chrysanthemi* (GenBank: Y00549), carrot (SwissProt Accession No. P83218) and *A. aculeatus* (Swiss-Prot code Q12535); and the PMEI domains in kiwi (SwissProt Accession number No. P83326) and *Arabidopsis* (*AthPMEI-1*, Accession Number NP_175236; *AthPMEI-2*, Accession Number NP_188348) were analyzed by T-Coffee (http://tcoffee.org/503/index.html) and displayed by ESPript 3.0 (http://espript.ibcp.fr/ESPript/cgi-bin/ESPript.cgi, Supplementary Figs. S9 and S10). The ZmPMEs contain five characteristic sequence fragments (44_GxYxE, 113_QAVAL, 135_QDTL, 157_DFIFG, 223_LGRPW; carrot numbering), and several highly conserved aromatic residues (Supplementary Fig. S9). The ZmPMEIs contain four conservative Cys residues, which were connected by two disulfide bridges (first to second and third to fourth) and do not have the fifth conservative Cys residue which has a free thiol group comparing to kiwi and *Arabidopsis*.

**Figure 4:**
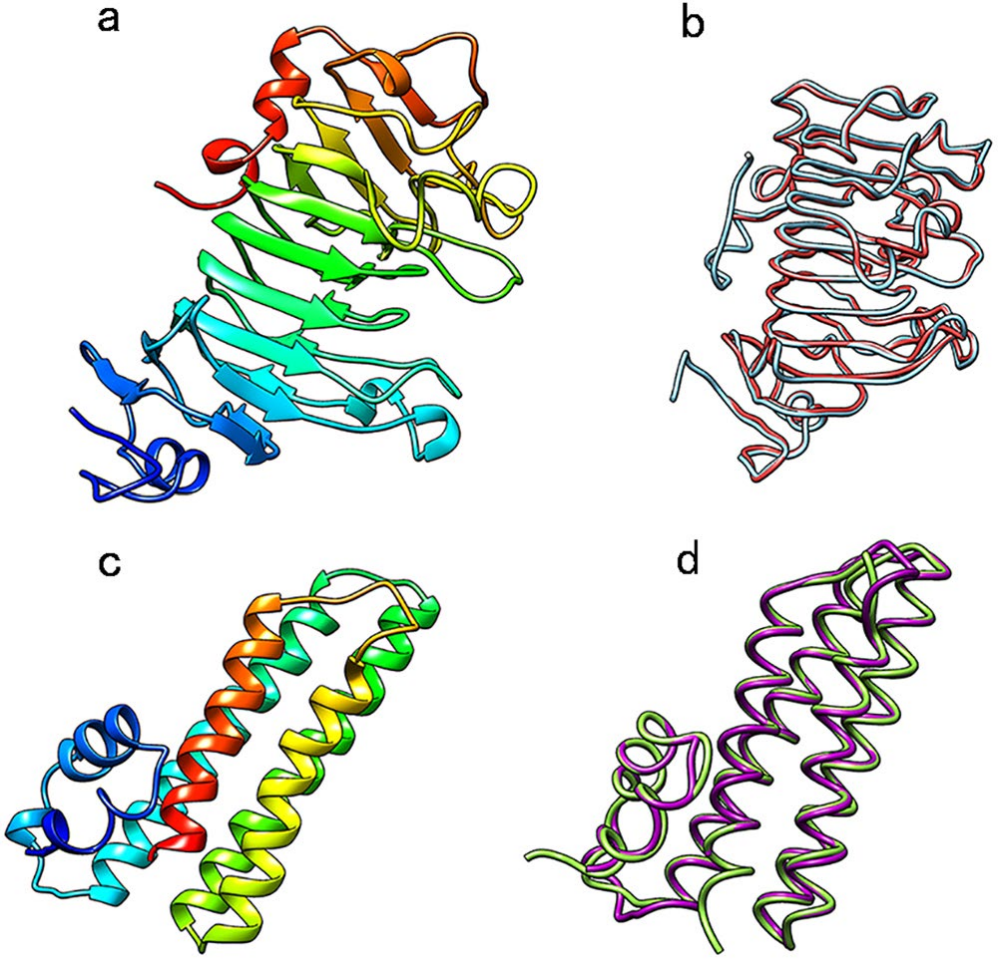
Structure of the PME/PMEI domains in two ZmPME/PMEIs. The 3D structure of PME/PMEI domains in ZmPME3 (**a**) and ZmPMEI2 (**c**). (**b**) Overlay of Cα trace of PME from maize (red) and PME from carrot (blue). Structures are almost completely superimposable, (C-score = 1.71, TM-score = 0.95, RMSD = 2.8). (**d**) Overlay of Cα trace of PMEI from maize (purple) and PMEI from kiwi (green), they high similarity (C-score = 1.71, TM-score = 0.95 ± 0.05, RMSD = 2.8 ± 2.0). The 3D structure of the PME/PMEI domains are developed using I-TASSER (http://zhanglab.ccmb.med.umich.edu/I‐TASSER/) and exhibited by Chimera1.8.1 (http://www.cgl.ucsf.edu/chimera/).

To further understand the structure of the ZmPME/PMEI proteins, three-dimensional (3D) structure of the PME/PMEI domains in ZmPME3 and ZmPMEI2 were analyzed by I-TASSER (https://zhanglab.ccmb.med.umich.edu/I-TASSER/), and exhibited by Chimera1.8.1 (http://www.cgl.ucsf.edu/chimera/, Fig. 4). ZmPME3 has high similarity with the PME (PDB 1GQ8) from Carrot^40^ (C-score = 1.71, TM-score = 0.95 ± 0.05, RMSD = 2.8 ± 2.0, Fig. 4b). Furthermore, ZmPMEI2 has high similarity with the PMEI (PDB 1xg2B) from kiwi^30^ (C-score = 1.7, TM-score = 0.86 ± 0.07, RMSD = 2.7 ± 2.0, Fig. 4d). Superposition of the known PME structures of carrot and maize (ZmPME3, Fig. 4b), and PMEI structures of kiwi and maize (ZmPMEI2, Fig. 4d) confirm the similarity of the folding topologies.

### Gene ontology (GO) annotation and subcellular localization of the ZmPME/PMEI proteins

**Figure 5:**
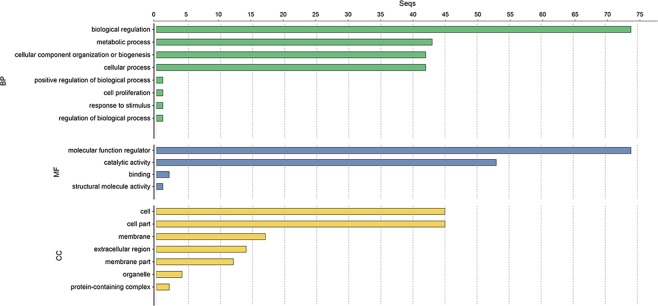
Gene Ontology (GO) analysis of the ZmPME/PMEI proteins. The ZmPME/PMEI amino acid sequences were annotated using the local Blast2GO program (https://www.blast2go.com/blast2go-pro/download-b2g). BP: biological process, MF: molecular function, CC: cellular component.

The 92 *ZmPME*/*PMEI* genes (except *ZmPME28*) were assigned a total of 37 GO terms (Fig. 5 and Table 1). Among them, 175, 78 and 167 proteins were assigned terms under molecular function, cellular component and biological process, respectively. Under biological process, 41 *ZmPME* genes were predicted to be involved in cell wall modification, 42 *ZmPME* genes were related to pectin catabolic process and 73 genes (all of *ZmPMEI* genes and *ZmPME21*, -*22*, -23 and -24) were involved in negative regulation of catalytic activity. Under cellular component, 41 *ZmPME* genes were assigned to cell part. Under molecular function, most of the *ZmPME* genes and a few *ZmPMEI* genes had pectinesterase activity and aspartyl esterase activity; and most *ZmPMEI* genes and a few *ZmPME* genes had pectinesterase inhibitor activity or enzyme inhibitor activity. In addition, we analyzed the GO annotations for each subfamily. The same annotations exist in different genes of different subfamilies (e.g., the *PMEI* genes in the subfamilies II and III), and there are also different annotations for genes in the same subfamily (e.g., the *PME* genes in the subfamily II, Supplementary Table 4). These results suggested that different genes in the same subfamily may have different roles in the evolution process.

**Table 1:** Gene ontology (GO) annotations of the *ZmPME*/*PMEI* genes.

Classification	GOs ID	Num	Annotation
Biological Process (167)	GO:0000079	1	regulation of cyclin-dependent protein serine/threonine kinase activity
	GO:0000723	1	telomere maintenance
	GO:0006281	1	DNA repair
	GO:0006310	1	DNA recombination
	GO:0006412	1	translation
	GO:0006508	1	proteolysis
	GO:0007088	1	regulation of mitotic nuclear division
	GO:0008152	1	metabolic process
	GO:0008284	1	positive regulation of cell proliferation
	GO:0032508	1	DNA duplex unwinding
	GO:0042545	41	cell wall modification
	GO:0043086	73	negative regulation of catalytic activity
	GO:0045490	42	pectin catabolic process
	GO:0045787	1	positive regulation of cell cycle
Cellular Component (78)	GO:0000307	1	cyclin-dependent protein kinase holoenzyme complex
	GO:0005576	13	extracellular region
	GO:0005618	41	cell wall
	GO:0005634	3	Nucleus
	GO:0005737	1	cytoplasm
	GO:0005840	1	ribosome
	GO:0005886	1	plasma membrane
	GO:0016020	4	membrane
	GO:0016021	12	integral component of membrane
	GO:0048046	1	Apoplast
Molecular Function (175)	GO:0003678	1	DNA helicase activity
	GO:0003735	1	structural constituent of ribosome
	GO:0004564	1	beta-fructofuranosidase activity
	GO:0004672	1	protein kinase activity
	GO:0004857	34	enzyme inhibitor activity
	GO:0005524	1	ATP binding
	GO:0008234	1	cysteine-type peptidase activity
	GO:0016538	1	cyclin-dependent protein serine/threonine kinase regulator activity
	GO:0016787	1	hydrolase activity
	GO:0019901	1	protein kinase binding
	GO:0030599	52	pectinesterase activity
	GO:0045330	41	aspartyl esterase activity
	GO:0046910	39	pectinesterase inhibitor activity

Subcellular localization of the 92 ZmPME/PMEIs were predicted using TargetP (http://www.cbs.dtu.dk/services/TargetP/) and WoLF PSORT (https://wolfpsort.hgc.jp/). Majority of the proteins (77, 83.7%) were revealed as signal peptides by TargetP; five (5.4%) are located in mitochondria; and ten are not assigned (Supplementary Table 1). Moreover, the WoLF PSORT predicted a number of ZmPME/PMEIs (93.5%) locating to chloroplast or extracellular (Supplementary Table 1). In addition, a *ZmPMEI* gene (*ZmPMEI16*) was found to be targeted to chloroplast by an *in vivo* transient expression assay (Supplementary Fig. S8). This consistent with the prediction of WoLF PSORT.

### Expression assay of the *ZmPME*/*PMEI* genes

The NimbleGen maize microarray data^41^ (ZM37) including 60 tissues representing 11 major organ systems and various developmental stages of the B73 maize inbred line was employed to analyze the expression pattern of the *ZmPME*/*PMEI* genes. All of the *ZmPME*/*PMEI* genes (except one *ZmPME* gene and 13 *ZmPMEI* genes) expression data was used to draw Heatmap. Of them, 35 *ZmPME*/*PMEI* genes had a much higher expression level in anthers than in other tissues (Fig. 2), these *ZmPME*/*PMEI* genes may be related to the development of anther or pollen. In general, expression pattern was similar for genes within the same paralogous gene pairs (e.g., *ZmPMEI1*/-17, Supplementary Table 1 and Fig. 2), indicating they might be formed by segmental duplication and retained their function. However, the expression profiles of the four paralogous gene pairs (*ZmPME12*/-13, *ZmPME14*/*-15*, *ZmPME16*/-17 and *ZmPMEI29*/-38) were fundamentally different in different tissues, suggesting that these genes may have differentiated with different roles.

**Figure 6:**
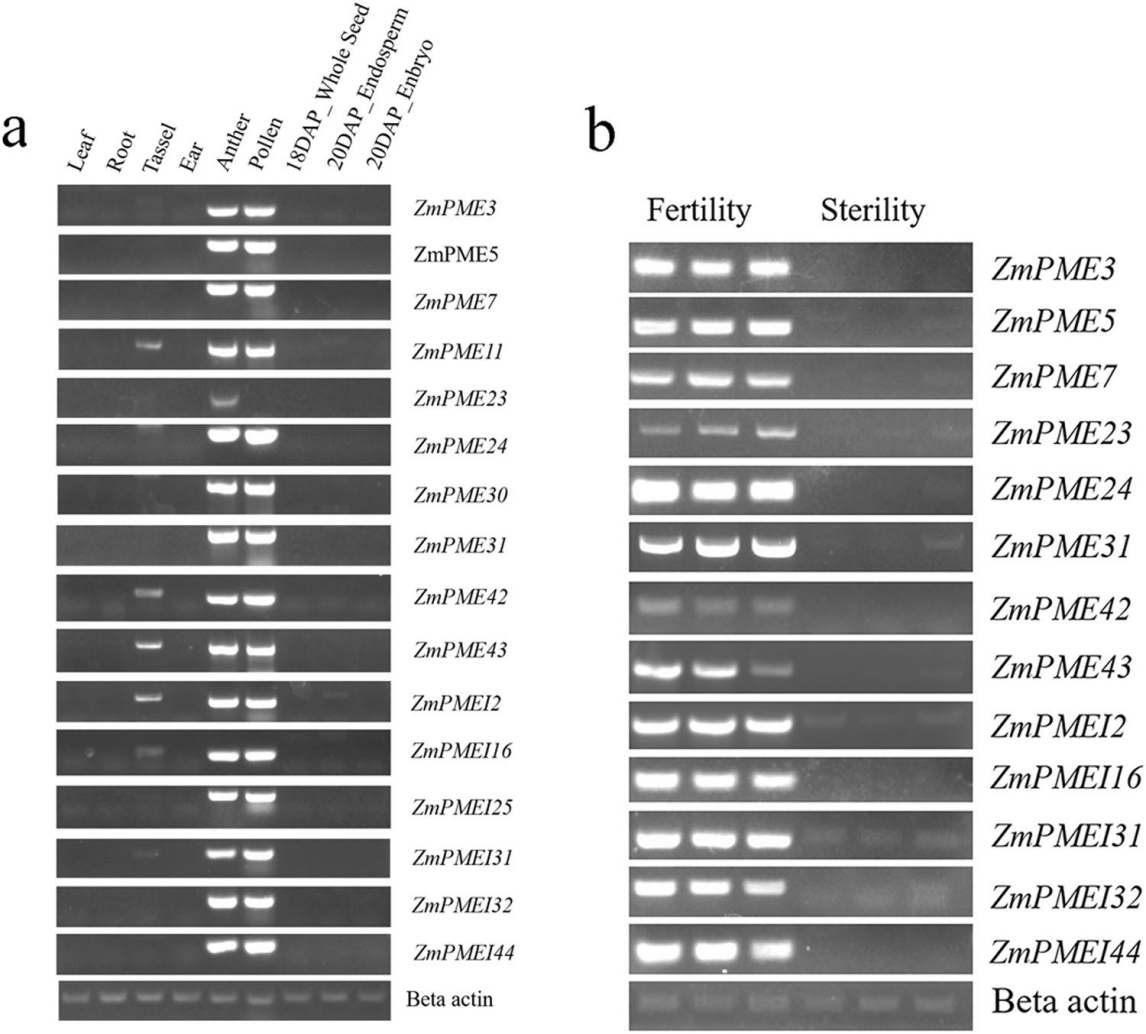
Semiq-RT-PCR analysis of some *ZmPME*/*PMEI* genes in the nine different tissues of B73 inbred line and in the anthers of three fertile and three sterile individuals of a maize backcross population. (**a**) The total RNA of nine tissues including seedling roots, seedling leaves, 2-cm immature ears, non-emerged immature tassels, anthers, pollen, whole seeds after pollination for 18 days, endosperm and embryo after pollination for 20 days of B73 inbred line was isolated and used to perform the semiq-RT-PCR of the *ZmPME*/*PMEI* genes. (**b**) The total RNA of anthers of three sterile and three fertile individuals was isolated and used to perform the semiq-RT-PCR of the *ZmPME*/*PMEI* genes. Beta actin was used for internal controls to normalize the RNA contents in each sample. Primers used are shown in Supplementary Table 5. In the figure, the PCR products were separated with the same experiment condition and that gels were processed in parallel. The original gels were presented in Supplementary Fig. S11.

To confirm the organ-specific expression of *ZmPME*/*PMEI* genes shown by the microarray data, 14 *ZmPME*/*PMEI* genes specifically expressed in anthers, *ZmPME24* (not including in the maize microarray data) and *ZmPME30* (expressed low in all tissues) were selected for conducting semiq-RT-PCR. Semiq-RT-PCR was performed with total RNA isolated from the roots, leaves, ears, immature tassels, pollens, anthers, whole seed (after pollinated), endosperm and embryo of the B73 inbred line. Fourteen of them, *ZmPME3*, -5, -7, -11, -23, -31, -42, -43 and *ZmPMEI2*, -16, -25, -31, -32, -44 matched well with the microarray data, all of these *ZmPME*/*PMEI* genes expressed significantly higher in the anthers or pollens than in other organs. It is interesting to note that *ZmPME23* was specifically expressed in the anthers. The gene not included in the microarray data, *ZmPME24*, was also specifically expressed in the anthers and pollens. However, expression of only one gene, *ZmPME30*, did not match with the microarray data, it had higher expression level in the anthers and pollens but showed little or no expression in roots, leaves, ears and seed according to the semiq-RT-PCR assay (Fig. 6a).

To further analyze the possible role of the *ZmPME*/*PMEI* genes on anther or pollen development, expression of the 13 genes (which had identified specifically expression in the anthers or pollens of B73 inbred line) was investigated in the anthers of three fertile and three sterile individuals of a maize backcrossing population derived from a cytoplasmic male sterility (CMS) line. The results showed that all of the selected *ZmPME*/*PMEI* genes were differentially expressed in the fertile and sterile anthers, although some *ZmPMEI* genes showed lower expression in the sterile anthers (Fig. 6b).

## Discussion

Genome-wide analysis has identified the PMEs and PMEIs in nearly all vascular plants and in multiple gene members^18^. Up to now, the function of a number of the *PME* genes have been studied in *Arabidopsis*^42^, rice^9^, pea^43^, wheat^44^, and cotton^45^; and the *PMEI* genes in *Arabidopsis*^46,47^, rice^13^, broccoli^48^, and Chinese cabbage^49^. Most of them involved in plant growth and various stress responses (reviewed by Wormit and Usadel^50^). In maize, however, only three ZmPMEs (*ZmC5*, *ZmPme3* and *ZmGa1P*) and one ZmPMEIs (*ZmPMEI1*) were characterized and found to be involved in pollen tube elongation^37,38,51,52^. Thus genome-wide identification, evolution, and expression analysis of the PME/PMEI families in maize will facilitate to understanding of the function of the gene families.

In this study, 43 and 49 ZmPMEs and ZmPMEIs were identified in the maize genome, which were divided into 5 and 8 subfamilies (Supplementary Table 1; Fig. 1), respectively. The number of ZmPMEs is less than that identified in *Arabidopsis*^7^, and *Gossypium raimondii*^11^, while that of ZmPMEIs is much more than that identified in *Sorghum bicolor*^12^, and *Brachypodium distachyon*^53^. We identified 24 paralogous pairs in maize, but all Ka/Ks values of paralogous are less than 1 (Supplementary Table 2), implying that *ZmPME*/*PMEI* genes evolved mainly under the influence of stabilizing selection. The result of Ka/Ks analysis of PME, PRO and PMEI domains reveals that the homologous gene pairs of *Arabidopsis*, rice and sorghum experienced purifying selection^12^ and the PME homologous gene pairs of *G*. *arboreum*, *G*. *raimondii* and *G*. *hirsutum* also experienced stabilizing selection^45^. Thus *ZmPME*/*PMEI* genes might play important role in growth and development of plants.

Intragroup ZmPME/PMEIs have conserved gene structure and motif composition, indicating that ZmPME/PMEIs in the same group could have the same function and they might come from a common ancestor. For instance, the ZmPME subfamilies IV and V contain motifs 1–5 (except *ZmPME32*, -34 and -43) and most intron phases 2, 0, 0 and 2 separating the PME domain (Fig. 3a,b). Of the ZmPMEIs, most members in the subfamilies III, IV and VIII expressed higher in anthers than in other tissues (Fig. 2), and most members in the subfamily VII have motifs 8 and 9 (Fig. 3c,d). The PME and PMEI domains alignment implying that the ZmPME domains have five characteristic sequence fragments (44_GxYxE, 113_QAVAL, 135_QDTL, 157_DFIFG, 223_LGRPW; carrot numbering, Supplementary Fig. S9), which have all been shown to be functionally important in carrot^54^; the ZmPMEI domains have four conservative Cys residues (Supplementary Fig. S10), which are connected by two disulfide bridges, but do not have the fifth conservative Cys residue in comparison with that in kiwi^29^ and *Arabidopsis*^55^. The structure of the carrot PME is almost completely superimposable to the structure of tomato^30^. In this study, the 3D structure of ZmPME3 and ZmPMEI2 are highly similar to that from Carrot^40^ and kiwi^29^, respectively. This indicated that the PME and PMEI domains were highly conserved in different plant species.

In recent years, there are many reports on the function of *PME*/*PMEI* genes. Overexpression of *PMEI5* in *Arabidopsis thaliana* caused aberrant growth morphology of the stems^56^; *VvPMEI1* expression negatively correlates with the PME activity during the early stage of grape berry development of Grapevine^57^. The expression pattern of the *ZmPME*/*PMEI* genes from the NimbleGen maize microarray data showed 35 *ZmPME*/*PMEI* genes had a much higher expression level in the anthers than in other tissues (Fig. 2), and semiq-RT-PCR analysis of different tissues from B73 inbred line verified that they had much higher expression in the anthers or pollens (Fig. 6a). In addition, semiq-RT-PCR analysis of some *ZmPME*/*PMEI* genes showed that 13 *ZmPME*/*PMEI* genes were differentially expressed in the anthers of fertile and sterile individuals derived from a maize S-type CMS line (Fig. 6b). Similar results also have been reported in other plant species. For example, antisense expression of a pollen-specific PMEI from broccoli (*Brassica oleracea*) in *Arabidopsis* triggered silencing of the orthologous *Arabidopsis* gene *At1g10770* and resulted in male sterility^48^; the expression of the pectin methylesterase gene (*At3g06830*) was significantly lower in male-sterile line than in male-fertile line at the <1 mm anther length stage of *Brassica napus*^58^; in cotton, transcriptome analysis showed that many pectin methylesterase genes highly expressed in flowering buds of fertile plants compared to those of the CMS-D8 line^59^. This implied that a number of *ZmPME*/*PMEI* genes might play an important role in anther and pollen development, however, their detailed roles in male function of maize need to be further studied in future.

## Materials and Methods

### Identification of the *PME*/*PMEI* genes in maize

Maize genome sequences were downloaded from the Maize Genome Database (Maize GDB; https://www.maizegdb.org/). Local HMMER3.0^39^ (E-value-10) searches were performed using the Hidden Markov Model (HMM) profile in the Pfam database (http://pfam.janelia.org/search/sequence) to screen all candidate ZmPME/PMEI gene sequences. Candidate genes were retained that contained known conserved domains and passed checks against the Pfam (http://pfam.janelia.org/) and SMART (http://smart.embl-heidelberg.de/) databases for presence of the PME/PMEI domains (PF01095/PF04043). Bioinformatics analyses were performed on the ZmPME/PMEI protein sequences, and physical and chemical parameters (e.g., MW, pI) were calculated using ExPASy (http://www.expasy.ch/tools/pi_tool.html). TargetP (http://www.cbs.dtu.dk/services/TargetP/) and WoLF PSORT (https://wolfpsort.hgc.jp/) were used to predict the subcellular of ZmPME/PMEIs.

### Analysis of gene structures and conserved motif of the *ZmPME*/*PMEI* genes

Several *ZmPME*/*PMEI* genes had more than one gene model annotated in MaizeGDB (https://www.maizegdb.org/). To confirm the putative alternative splicing transcripts, transcript-specific primers (Supplementary Table 5) were designed to amplify corresponding DNA isolated from B73 seedlings and cDNA derived from B73 pollen RNA. Conserved PME/PMEI domains and gene structures producing validated transcripts were drawn and displayed using the Gene Structure Display Server^60^ (GSDS2.0; http://gsds.cbi.pku.edu.cn/index.php).

Sequence alignment of ZmPME/PMEIs domain was conducted by T-Coffee (http://tcoffee.org/503/index.html), 3D structures were predicted through I-TASSER^61^ (http://zhanglab.ccmb.med.umich.edu/ITASSER/) and visualized by Chimera1.13.1 (http://www.cgl.ucsf.edu/chimera/).

### Phylogenetic and multiple alignment analyses

The PME/PMEI protein sequences were aligned using ClustalX 2.0^62^ (http://www.clustal.org/clustal2/) with the default parameters. Phylogenetic tree was drawn with the neighbor-joining method using software MEGA7.0^63^ (molecular evolutionary genetics analysis, https://www.megasoftware.net/) using pairwise deletion; 1,000 replicates were used for bootstrap analysis and the cut-off value was 50%. The information of *PME*/*PMEIs* genes of rice and *Arabidopsis* were obtained from two report of Yang *et al*.^9^ and Wang *et al*.^12^, and the protein sequences were downloaded from the *Arabidopsis* Information Resource (TAIR; https://www.arabidopsis.org/) and the Rice Genome Annotation Project websites (http://rice.plantbiology.msu.edu/analyses_search_locus.shtml).

We used the MEME system (http://meme.sdsc.edu/meme/itro.html) to identify conserved motifs with parameters set as: number of repetitions, arbitrary; maximum number of patterns, 20; optimal width of the motif, between 6 and 50 residues^64^.

### Calculation of synonymous (Ks) and non-synonymous (Ka) substitutions

To identify homologous pairs of genes, the transcript sequences of the ZmPME/PMEIs were investigated by BLASTN searches^65^. Paralogous pairs within the genome of maize were defined as follows: the aligned sequences were longer than 300 bp and shared identities ≥40%^66^. If the amino acid shorter than 300 bp, the aligned region had an identity ≥60% and the alignment length covered ≥50% of the gene were defined paralogous pairs.

### Gene ontology (GO) annotation

The translated ZmPME/PMEIs protein sequences were annotated using the Blast2GO5.2.4 program to assign GO terms^67^ (http://amigo.geneontology.org/amigo/term/). GO analysis e-value is 1.0E-6. GO terms are provided under three main categories, biological process, cellular component, and molecular function.

### Localization of fluorescent protein-tagged ZmPMEI16

Full-length ORF of ZmPMEI16 was isolated by PCR using primers ZmPMEI16-pM999-F (5′-AGCAGATCTATCGATGAATTCATGGGGCAAGCCTACCCA-3′) and ZmPMEI16-pM999-R (5′-TCCTTTGCCCATGGCTCTAGATCATATCATGTTTGCAAGCG-3′). The resulting fragment was digested with *EcoRI* and *XbaI* and inserted between the corresponding sites of pM999-EGFP (provided by professor Liwen Jiang), which express an engineered version of the green-fluorescent protein (GFP) under the control of the cauliflower mosaic virus 35S promoter. The plasmid pZmPMEI16-GFP and pM999-EGFP were used for transient expression experiments in maize protoplasts^68^. Samples were analyzed by confocal laser scanning microscopy using a Leica TCS-SP8 operating system as described by Ravanel *et al*.^69^.

### Expression analysis of the *ZmPME*/*PMEI* genes in different tissues

To investigate the spatiotemporal expression patterns of the *ZmPME*/*PMEI* genes, RMA-normalized data for *ZmPME*/*PMEI* genes were downloaded from PLEXdb (http://www.plexdb.org/). A heat map was produced by Heml 1.0.3.7- Heatmap illustrator.

### Semi-quantitative reverse transcription PCR (semiq-RT-PCR)

Total RNA of the B73 inbred line was extracted using the Trizol reagent (Invitrogen, USA) according to the manufacturer’s recommendations. In addition, anthers on tassels (about to exsert from the upmost leaves) were collected from sterile and fertile plants of a backcrossing population derived from a maize S-type CMS, and RNA was also extracted using the same method. First-strand cDNA was synthesized from 0.05–5 μg of total RNA (20 μL reaction volume) using *TransScript* First-Strand cDNA Synthesis Super Mix (TransGen Biotech). All gene-specific primers were designed by primer 3 (http://primer3.ut.ee/) as shown in Supplementary Table 5. The maize gene Actin1 (GenBank ID: NM_001155179) was used as an internal control. The semiq-RT-PCR assays were repeated for two or three times (biological replications).

## Supplementary information

Supplementary information

Supplementary information 2
