# Supplementary material for: Genome-wide identification, phylogeny and expression analysis of the PME and PMEI gene families in maize
Source: Sci Rep. 2019 Dec 27;9:19918. doi: 10.1038/s41598-019-56254-9 (PMC6934449; doi:10.1038/s41598-019-56254-9)
Supplement: Supplementary file 1 — Supplementary information [file 41598_2019_56254_MOESM1_ESM.pdf]

Genome-wide identification, phylogeny and expression analysis of the PME and PMEI gene families in maize

Panpan Zhang[1], Hao Wang[1], Xiner Qin[1], Kuan Chen[1], Jiuran Zhao[2], Yanxin Zhao[2,*], Bing Yue[1,*]

[1] National Key Laboratory of Crop Genetic Improvement, Huazhong Agricultural University, Wuhan 430070, China

[2] Beijing Key Laboratory of Maize DNA Fingerprinting and Molecular Breeding, Maize Research Center, Beijing Academy of Agriculture and Forestry Sciences, Beijing 100097, China

[*] **Corresponding author:**

Yanxin Zhao

Email: rentlang2003@163.com

Phone: +86-01051503536

Bing Yue

Email: yuebing@mail.hzau.edu.cn,

Phone: +86-27-87286870

Supplementary Figures

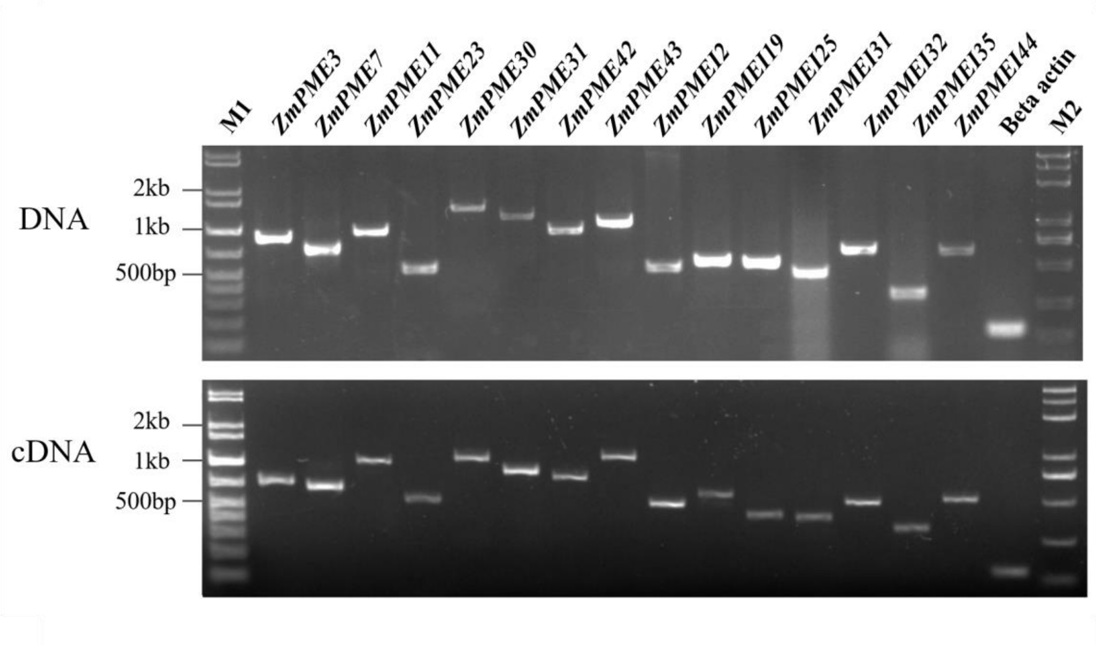

Supplementary Figure 1. Confirmation of gene structure of the *ZmPME*/*PMEI* genes by RT-PCR. DNA of seedlings and cDNA of pollens in maize inbred B73 were used as templates to amplify the *ZmPME*/*PMEI* genes with the primers listed in Supplementary Table S5. The PCR products were analyzed in 1.5% agarose gel. M1: 1Kb plus DNA ladder (TIANGEN BIOTECH); M2: Trans2K plus DNA Marker (TransGen Biotech).

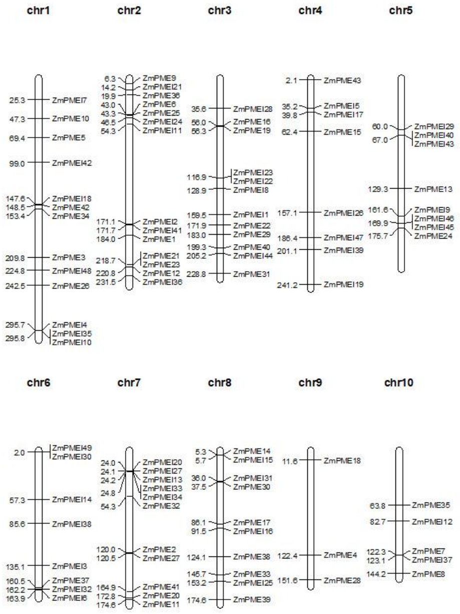

Supplementary Figure 2. Chromosomal location of the *ZmPME*/*PMEI* genes. All of the 92 maize *PME*/*PMEI* genes mapped onto the maize chromosomes based on their annotation data from MaizeGDB (http://www.maizegdb.org/) and displayed by Map Chat2.3[1].

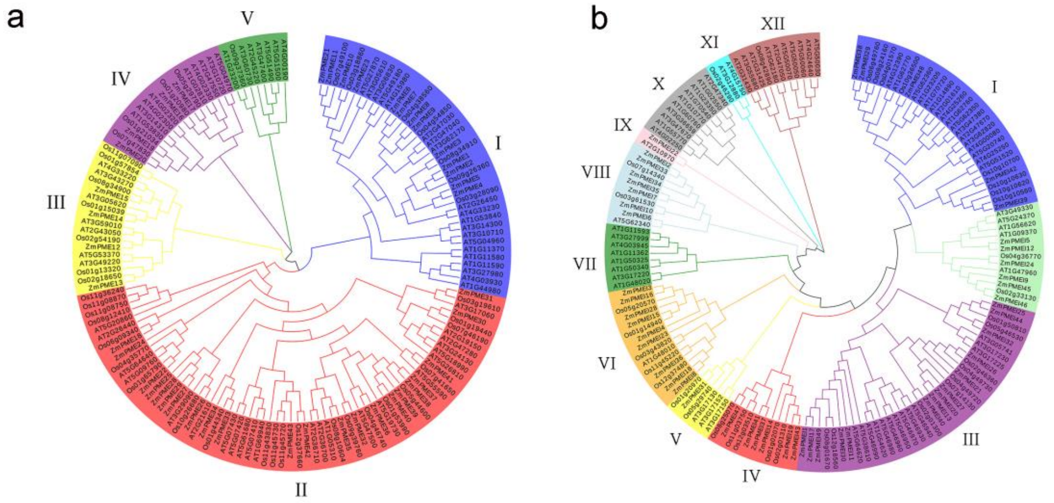

Supplementary Figure 3. Phylogenetic trees of PMEs and PMEIs from maize, rice and *Arabidopsis*. (a) Phylogenetic tree of PMEs. (b) Phylogenetic tree of PMEIs. The phylogenetic trees are based on a sequence alignment of the PMEs or PMEIs protein sequences from maize, rice and *Arabidopsis*. The PMEs and PMEIs were grouped into 5 (I-V) and 12 (I-XII) distinct clades, respectively. The phylogenetic trees were built by MEGA7 (https://www.megasoftware.net/).

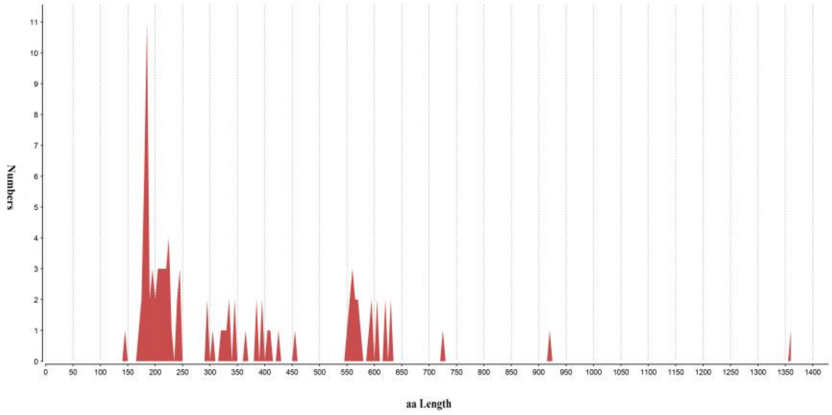

Supplementary Figure 4. Distribution of the length of the ZmPME/PMEIs. The full-length protein of the *ZmPME*/*PMEI* genes were drawn by Blast2GO5.2.4[2].

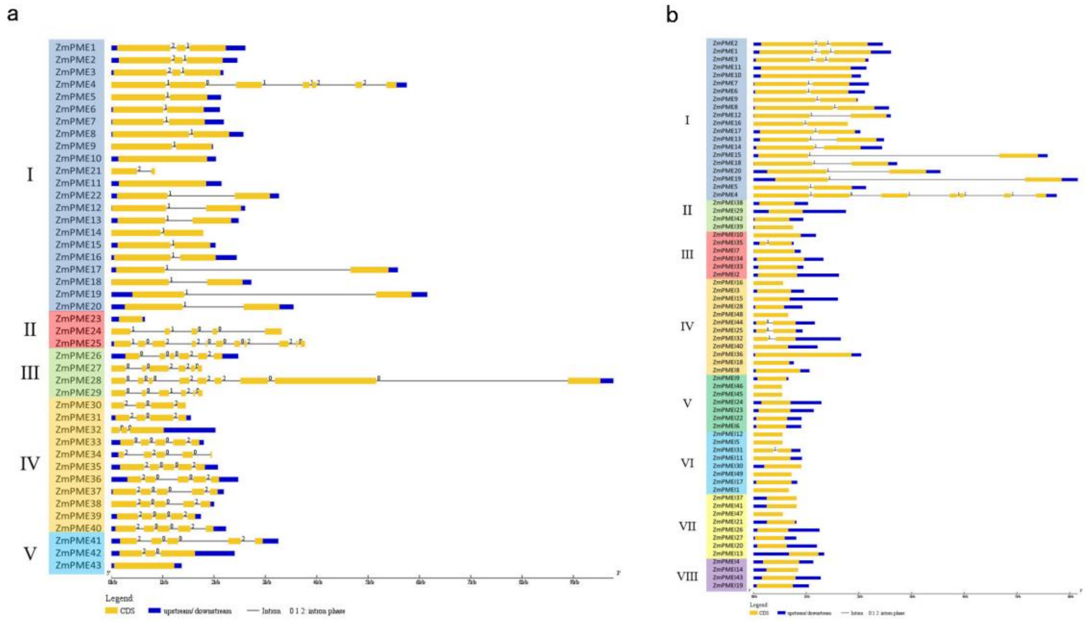

Supplementary Figure 5. Genes structure of the *ZmPME*/*PMEI* genes. The gene structures of the *ZmPME* genes (a) and *ZmPMEI* genes (b) were built using GSDS2.0[3] (http://gsds.cbi.pku.edu.cn/index.php) through both alignment of DNA obtained from MaizeGDB (http://www.maizegdb.org/) and coding sequences (CDS) of the *ZmPME*/*PMEI* genes.

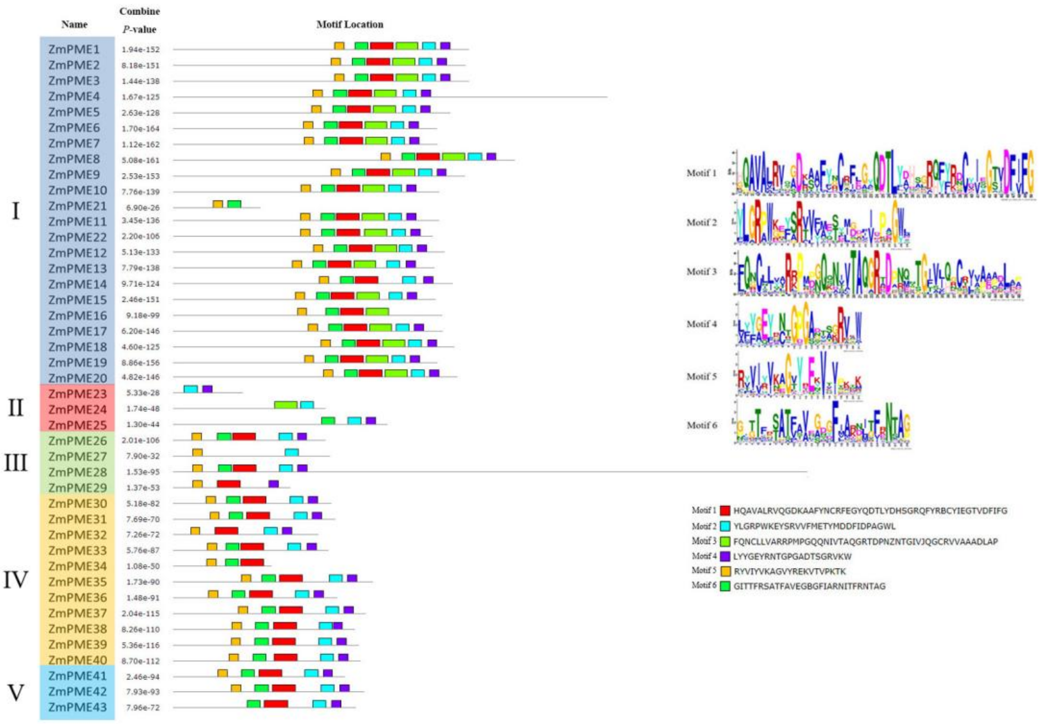

Supplementary Figure 6. Schematic representation of 6 conserved motifs in the *ZmPME* genes. Using the online MEME program[4] (http://meme.sdsc.edu/meme/itro.html), conserved motifs in the *ZmPME* genes were identified. Different colored boxes represent different specific motifs.

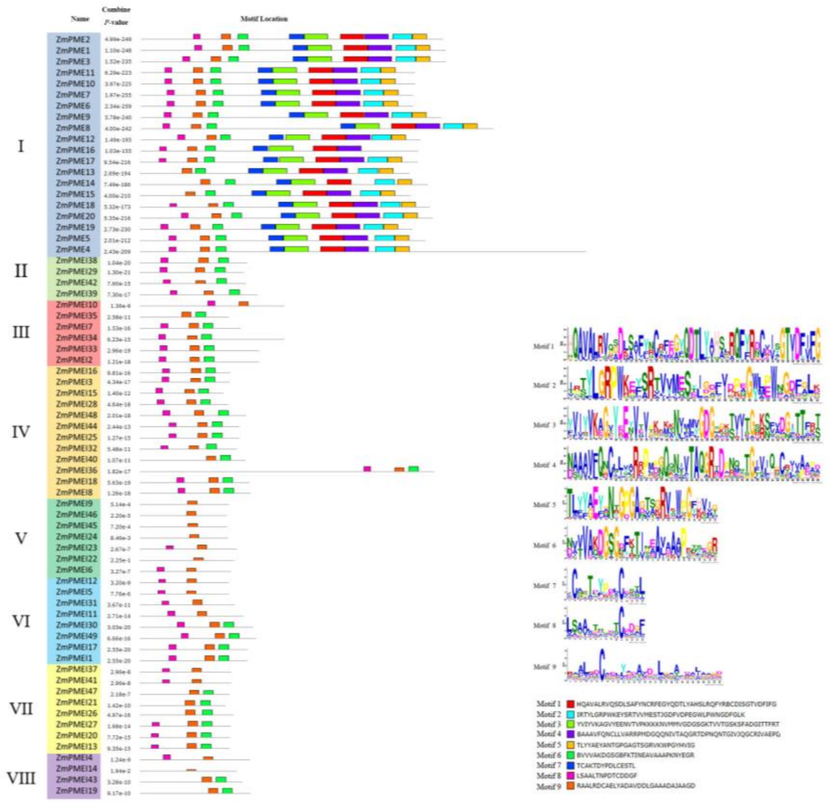

Supplementary Figure 7. Schematic representation of 9 conserved motifs in the *ZmPMEI* genes. Using the online MEME program[4] (http://meme.sdsc.edu/meme/itro.html), conserved motifs in the *ZmPMEI* genes were identified. Different colored boxes represent different specific motifs.

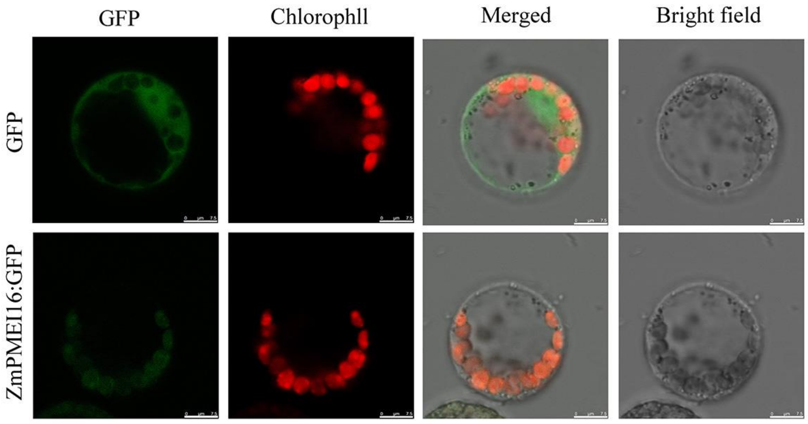

Supplementary Figure 8. Targeting of ZmPMEI16 to the chloroplast. The ZmPMEI16:GFP was expressed in maize protoplasts and their subcellular location was examined by laser confocal microscopy. Signals from GFP fluorescence (green), chlorophyll autofluorescence (red) and the merged and bright field images are shown. Bar 7.5 μm.

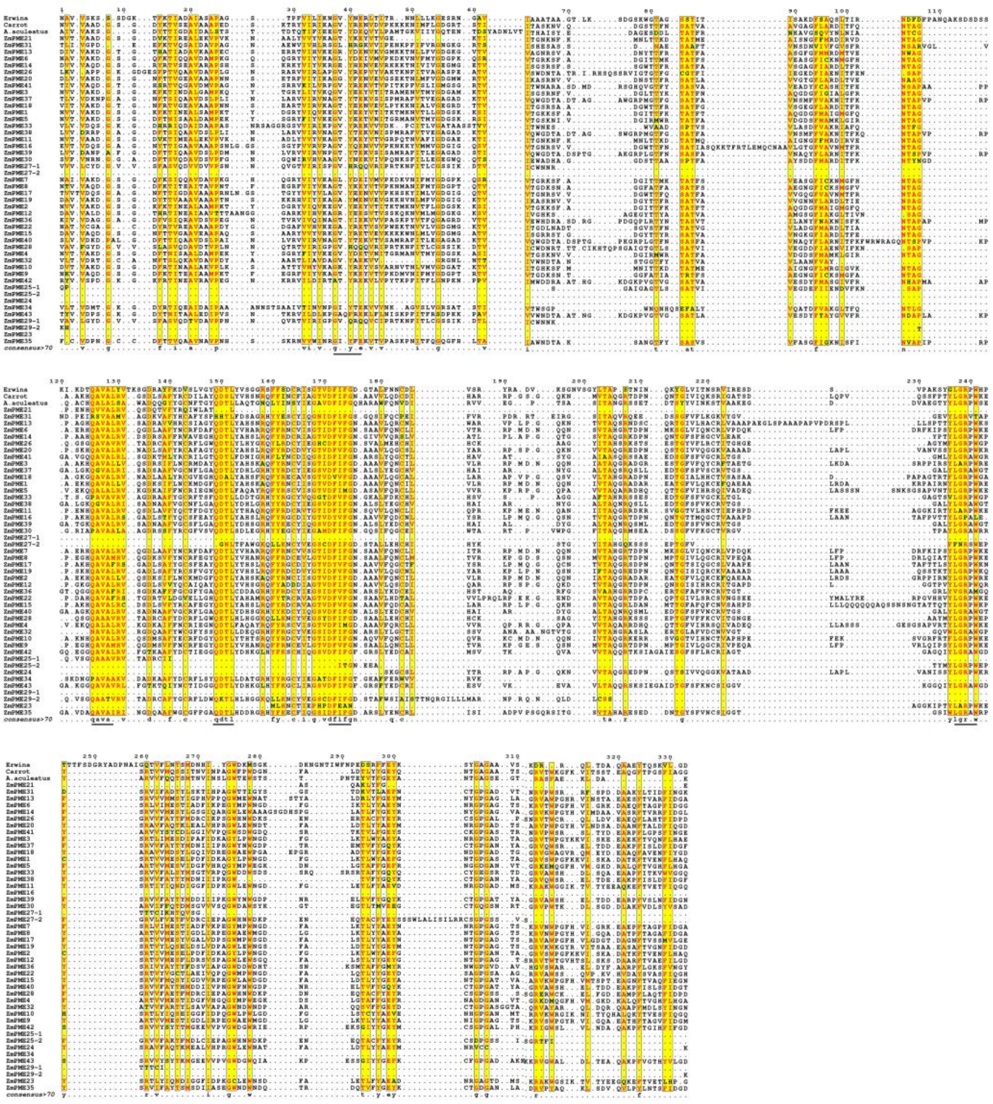

Supplementary Figure 9. Similarity of PME domains in the ZmPMEs. Multiple sequence alignment was conducted by T-Coffee (http://www.tcoffee.org/), and displayed by ESPript 3.0[5] (http://espript.ibcp.fr/ESPript/cgi-bin/ESPript.cgi). The yellow amino acid indicates the conservative sequences. The underline amino acid indicates five characteristic sequence fragments (44_GxYxE, 113_QAVAL, 135_QDTL, 157_DFIFG, 223_LGRPW; carrot numbering). ZmPME25 (or 27, 29)-1 and ZmPME25 (or 27, 29)-2 indicates that it has 2 PME domains.

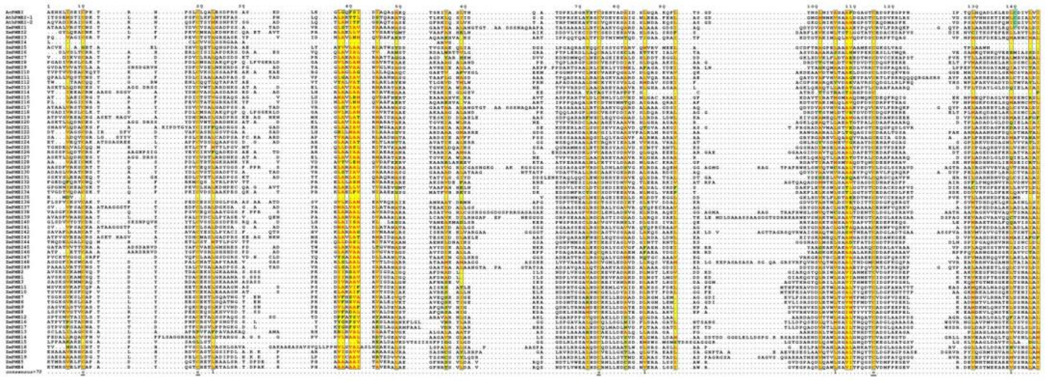

Supplementary Figure 10. Similarity of PMEI domains in the ZmPMEIs. Multiple sequence alignment was conducted by T-Coffee (http://www.tcoffee.org/) and displayed by ESPript 3.0[5] (http://espript.ibcp.fr/ESPript/cgi-bin/ESPript.cgi). The yellow amino acid indicates the conservative sequences. The underline amino acid indicates four conservative Cys residues, which are connected by two disulfide bridges (first to second and third to fourth), the green amino acid indicates fifth conservative Cys residue (which has a free thiol group) in other species.

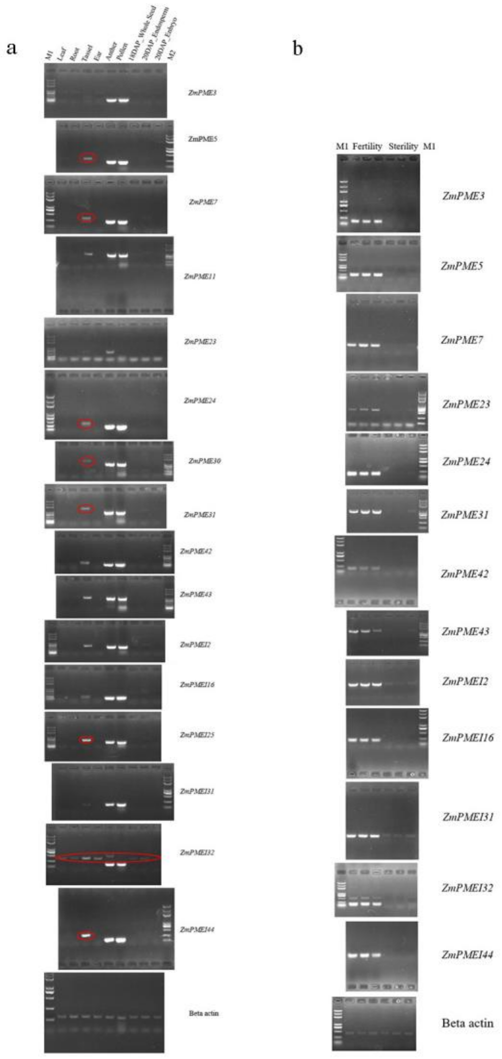

Supplementary Figure 11. The original gels of ‘Figure 6’. (a) The original gels of ‘Figure 6a’. (b) The original gels of ‘Figure 6b’. M1 and M2 were DNA Markers (Trans2K plus DNA Marker from TransGen Biotech). The circles in red represent nonspecific amplicons. DNA Markers located in the middle of a big gel.

References

1. Voorrips, R.E. MapChart: Software for the Graphical Presentation of Linkage Maps and QTLs. *J Hered*. **93**: 77-78 (2002).
2. Conesa, A., Götz, S. Blast2GO: A comprehensive suite for functional analysis in plant genomics. *Int J Plant Genomics*. **2008**: 619832 (2008).
3. Hu, B., Jin, J., Guo, A.Y., Zhang, H., Luo, J., Gao, G. GSDS 2.0: an upgraded gene feature visualization server. *Bioinformatics*. **31**: 1296 (2014).
4. Bailey, T.L., Williams, N., Misleh, C., Li, W.W. MEME: discovering and analyzing DNA and protein sequence motifs. *Nucleic Acids Res*. **34**: 369-373. (2006).
5. Robert, X., and Gouet, P. Deciphering key features in protein structures with the new ENDscript server. *Nucleic Acids Res*. **42**: W320-W324 (2014).
